# A Further Response to Shah Ebrahim

**DOI:** 10.1371/journal.pmed.0020189

**Published:** 2005-07-26

**Authors:** Michael Makover

**Affiliations:** **1**New York University School of MedicineNew York, New YorkUnited States of America

Shah Ebrahim says, in his answer to my statement in our debate [1], that plaque is so common that it makes sense to treat only those likely to have an acute event in the near future. Not everyone has a heart attack but everyone ages, nor can we be sure who will be lucky as they age and who will not. Anyone with a low-density lipoprotein level above 70 mg/dl is at risk [2]. Narrowing of arteries must certainly contribute to health decline in aging. He says that most plaque is stable and does not rupture. The half-million people who have a heart attack and the hundreds of thousands of individuals who have a stroke in the United States alone each year would disagree. Waiting decades more for further studies will not help all those now succumbing to the disease, as long as all the components have already been well vetted, as they have been. Ebrahim's statement that “most [plaques] are stable and unlikely to rupture” is based on his own study published in *Stroke* [3], but the paper has no data relating to how many patients found to have increased intima-media wall thickness (IMT) or plaque actually had heart attacks or strokes. It did find that intimal-wall changes were highly associated with ischemic heart disease.

It is correct that risk factors can predict heart disease and stroke to some degree, though I have many patients for whom that approach fails while IMT detects the risk otherwise missed. However, risk factor analysis is not enough. Changes in smoking, diet, weight, blood pressure, and such are all targets for treatment, but how do doctors know whether they have controlled them adequately? If IMT increases, more control is required. If IMT stabilizes or reverses a little, then it means that control is at goal. Family history of premature coronary artery disease is a major but underused risk factor. The causative factors have not yet been elucidated by research, so we do not know what the goals are. However, IMT provides a highly satisfactory parameter by which to judge the effectiveness of treatment in familial coronary artery disease: IMT stabilization and reversal are good, whereas progression is bad and requires more intensive measures.

Epidemiology and public-health planning correctly look at policies to apply to large populations. However, the practice of medicine is patient by patient, accomplished in the face-to-face doctor–patient relationship. Policy can be useful as a resource, but each patient should have the maximum individualized care and access that a doctor can provide. Patients should be able to make their own informed choices and not be dictated to by policies meant for masses. Shouldn't everyone with increased IMT at least be informed of the options available to limit it rather than waiting until acute events or advanced narrowing occur? It would be best to start a healthy lifestyle from birth, but fortunately by adulthood there is still time to make an enormous difference in practical terms if we take action at the stage when intimal widening is detectable with the highly sensitive ultrasound described in my original viewpoint [1].
